# Effect of *Enterococcus faecium* as a Water and/or Feed Additive on the Gut Microbiota, Hematologic and Immunological Parameters, and Resistance Against Francisellosis and Streptococcosis in Nile Tilapia (*Oreochromis niloticus*)

**DOI:** 10.3389/fmicb.2021.743957

**Published:** 2021-10-01

**Authors:** Suelen Aparecida Suphoronski, Felipe Pinheiro de Souza, Roberta Torres Chideroli, Leonardo Mantovani Favero, Natália Amoroso Ferrari, Henrique Momo Ziemniczak, Daniela Dib Gonçalves, Nelson Mauricio Lopera Barrero, Ulisses de Padua Pereira

**Affiliations:** ^1^Fish Bacteriology Laboratory, Department of Preventing Veterinary Medicine, State University of Londrina, Universidade Estadual de Londrina, Londrina, Brazil; ^2^Postgraduate Program in Animal Science With Emphasis on Bioactive Products, Department of Veterinary Medicine, Universidade Paranaense, Umuarama, Brazil; ^3^Laboratory of Center for Study and Research in Aquaculture and Genetics, Department of Animal Science, State University of Londrina, Universidade Estadual de Londrina, Londrina, Brazil

**Keywords:** *Enterococcus faecium*, immunological parameters, routes of administration, tilapia, resistance to diseases

## Abstract

In the present study, we evaluated the effects of administering *Enterococcus faecium* in food and/or water on the hematological and immunological parameters, intestinal microbiota, resistance to bacterial diseases (streptococcosis and francisellosis) and growth of Nile tilapia. Before the *in vivo* experiment, probiotic bacteria isolated from Nile tilapia were selected *via* inhibition tests. Sequencing, annotation, and assembly of the complete genome of the selected bacteria as well as other tests were performed using bioinformatics tools. Three treatments were implemented: G1 (probiotic feeding), G2 (probiotic in water), and G3 (probiotic in food and water); and a negative control (NC) was also employed. Treatment lasted 38 days, and each group consisted of fish and two repetitions. The fish were divided and infected with *Streptococcus agalactiae* S13 (serotype Ib) and *Francisella orientalis*. The G1 group had a higher average final weight gain than the G2, G3, and NC groups. Further, a significant increase in the number of thrombocytes was observed in the groups administered probiotics in the diet (G1 and G3). A statistical difference was observed in the mortality of fish infected with *S. agalactiae* in the NC compared to the treated groups. *Cetobacterium* was the 43 most abundant genus in the intestinal microbiota of all groups, including the NC group. *E. faecium* increased the immunity of fish administered the treatment and decreased the mortality caused by *S. agalactiae*. As an autochtone probiotic, *E. faecium* does not interfere with the local ecosystem and thus has a great probiotic potential for Nile tilapia in Brazil.

## Introduction

Fish farming is one of the largest aquaculture activity in the world, with Nile tilapia (*Oreochromis niloticus*) being a species of great importance [Food and Agriculture Organization (FAO), 2020]. It is estimated that the culture of this species moves the economy of 135 countries worldwide, and in Brazil, this species is the most produced fish [Associação Brasileira de Piscicultura-Peixe-BR, 2020; Food and Agriculture Organization (FAO), 2020].

Brazil is estimated to lose 84 million dollars per year in freshwater fish farming owing to losses caused by diseases, with bacteriosis being the most important contributing disease (Tavares-Dias and Martins, 2017). Bacteriosis, such as streptococcosis and francicellosis, is known to cause high mortality rates in fish (Soto et al., 2009). Antimicrobials are used as treatment and prophylaxis for these diseases; however, the indiscriminate use of antibiotics promotes the selection of drug-resistant or multi-resistant bacteria, in addition to their potential risk to the environment and public health (Merrifield et al., 2010; Ruiz et al., 2020).

Therefore, the use of probiotics as an alternative strategy has been receiving increasing attention worldwide for tilapia aquaculture (Hai, 2015; Alemayehu et al., 2018; Bharati et al., 2019). Probiotics are live microorganisms that, when administered in adequate amounts, confer health benefits to the host. Several microorganisms, including Gram-negative and Gram-positive bacteria, have been used as probiotics in fish farming, including *Lactobacillus*, *Lactococcus*, *Leuconostoc*, *Enterococcus*, *Carnobacterium*, *Shewanella*, *Bacillus*, *Aeromonas*, *Vibrio*, *Enterobacter*, *Pseudomonas*, *Clostridium*, and *Saccharomyces* species (Nayak, 2010; Hai, 2015). Probiotics, either alone or combined with supplements, can elevate phagocytic, lysozyme, complement, respiratory burst activity, and the expression of various cytokines in fish (Wang et al., 2008, 2017; Ruiz et al., 2020). They also stimulate the gut immune system of fish, with a marked increase in the number of Ig (+) cells and acidophilic granulocytes (Nayak, 2010).

Studies have shown that the efficacy of probiotics is highest in the host species from which they are isolated. This is because the strains can perform better as they have already adhered to the gut wall of the fish and are well adapted to compete with the pathogens (Ghosh et al., 2010).

Nile tilapia fingerlings supplemented with *Bacillus cereus* for 42 days *via* water and feed had a significant increase in lysozyme, in addition to other immunological parameters. However, the results were better when the probiotic was added to the feed (Wang et al., 2017). Based on studies that administered *Lactobacillus plantarum* to Nile tilapias, the fish had higher feed efficiency, yield, and final weight after 12 weeks. Further, there was an increase in thrombocytes and leukocytes in these animals (Jatobá et al., 2011). In Nile tilapia, the use of commercial probiotics after 6 weeks positively affected fish zootechnical performance, increasing the number of goblet cells in the gut and the expression of immunity-related genes (Standen et al., 2016).

Most studies on probiotics isolated from other species or geographic regions may interfere with their mode of action. When the probiotic is isolated from the host itself, the chances of colonization/adhesion and its beneficial effects increase. In this context, the objective is to develop alternative methods for rearing tilapia to avoid the high use of antibiotics. The present study sought to investigate the use of *Enterococcus faecium* as a probiotic supplemented in the diet and water of *O. niloticus* to improve fish health and determine the effects of this probiotic on the intestinal microbiome, growth rates and zootechnical parameters of *O. niloticus*.

## Materials and Methods

### Probiotic Bacteria Selection, Genome Assembly, and Identification of Metabolic Regions

The *E. faecium* strain, LAC7.2, which was selected as a probiotic, was isolated from the gastrointestinal tract of healthy Nile tilapias from the hatchery of Londrina/Paraná, Brazil in 2017. *In vitro* tests were performed to suggest the probiotic potential. Nile tilapia feces were diluted (scale 10) in 0.85% saline, plated on Man, Rogosa, and Sharpe (MRS) Lactobacillus Kasvi^®^ agar, and incubated for 48 h at 28°C. Thereafter, colonies were selected and characterized. The selected bacteria strains were then seeded on MRS agar and incubated for 48 h. A solution containing Mueller Hinton agar (Kasvi, São José dos Pinhais, Brazil) at 45°C with pathogenic bacteria (*Escherichia coli*, *Staphylococcus* spp., and *Streptococcus* spp.) was prepared. This solution was placed on MRS plates containing probiotic bacteria. The plates were incubated for 24 h at 28°C, and the inhibition halos were measured for bacteria with larger halos. To evaluate the inhibition induced by *Francisella noatunensis* F1, cystine heart agar enriched with 1% of bovine hemoglobin (Kasvi) was employed, where the pathogenic bacteria were seeded with swabs over the plate surface. Thereafter, small holes were made in the agar and the *E. faecium* LAC 7.2 filtered supernatant (0.22 μm) was deposited. Readings were taken after 48 h. This test was performed in duplicate. The strain that shown the higher inhibition halo against pathogenic bacteria was considered as a potential probiotic for *in vivo* trial.

Genome sequencing of *E. faecium* was performed using the MiSeq platform (Illumina^®^, United States). Reads were uploaded in FASTQ format to the CLC Genomics Workbench 12 (Qiagen, United States) software for the trimming and assembly steps. Genome annotation was performed in Rapid Annotation using Subsystem Technology (RAST; version 2.0) (Aziz et al., 2008). The genome project was deposited in the GenBank database under the accession number CP045012.1. A summary of the project information is provided in Table 1.

**TABLE 1:** Genome sequencing project information, and *Enterococcus faecium* genome annotated by NCBI Prokaryotic Genome Annotation Pipeline (PGAP).

**Property**	**Term**
Finishing quality	Finished
Libraries used	One paired-and library (mean size 300 bp, DNA insert size of ∼300 bp)
Sequencing platforms	Illumina MiSeq
Fold coverage	377.0x
Assemblers	NCBI Prokaryotic workbench v12.0.2
Gene calling method	NCBI Prokaryotic Genome Annotation Pipeline
GeneBank ID	CP045012.1 (chromosome)
	CP045013.1 (plasmid pI)
	CP045014.1 (plasmid pII)
GenBank date of release	October 18, 2019
BIOPROJECT	PRJNA224116
Source material identifier	LAC7.2
Project relevance	Fish

**Attribute**	**Value**

Gene (total)	2.931
CDs (total)	2.841
Genes (coding)	2.710
Genes (RNA)	90
rRNAs	6, 6, 6 (5S, 16S, 23S)
rRNAs	68
ncRNAs	4
Pseudogenes (total)	131

Phylogenetic analysis was performed using Gegenees V2.2.1 (Ågren et al., 2012) and SplitsTree4 v4.15.1, with high accuracy, to generate a heatmap and phylogenetic tree, respectively. Secondary metabolite clusters in the sequenced genome were predicted using antiSMASH 5.0 (Weber et al., 2015) and RAST. The ResFinder 3.2 program was also used to detect resistance genes.

### Fish

A total of 405 Nile tilapia (*O. niloticus*) were obtained from a commercial hatchery in the state of Paraná, with an initial weight of 11.93 ± 0.59 g. The animals were stored in 150 L tanks containing heated water with continuous renewal (80% of daily volume) for an acclimatization period of 7 days; the temperature was maintained at approximately 25°C and fish were fed three times per day until apparent satiety. Water parameters (pH, 6.8–7.2; total ammonia, <0.4 mg/L, dissolved oxygen, 5.4 mg/L; and absence of chlorine) were measured daily and maintained throughout the experimental period. The microbiological diagnosis was performed before the experiment, where 20 fish were randomly sampled and killed using a high dose of benzocaine (200 mg/mL). All animal procedures were approved by the Ethics Committee on Animal Use of the State University of Londrina (CEUA/UEL-7327.2017.39). Fragments of the brain, liver, cranial kidney, and spleen were stripped in Mueller Hinton agar enriched with 5% defibrinated sheep blood and in cystine heart agar enriched with 1% of bovine hemoglobin. The plates were incubated at 28°C for 5 days to confirm the health status of the fish (no bacterial growth in the plates).

### Experimental Design and Basal Diet

Fish were divided into three treatment groups: G1 (probiotic rationing), G2 (probiotic in water), and G3 (probiotic in feed and water); and three control groups: PCA (no probiotic and challenge with *Francisella orientalis*), PCB (no probiotic and challenge with *Streptococcus agalactiae*), and NC (no probiotic and no challenge) (*n* = 45), as shown in Table 2. The fish were fed a commercial feed (Presence^®^ Nutripiscis TR 36% CP) according to the treatments and challenged on day 38. After infection, the animals were monitored for 30 days to determine clinical signs and mortality.

**TABLE 2:** Division of groups before and after challenge.

**Before challenge**		
**Group**	**Treatment**	**Tanks**

G1	Probiotic in feed	2
G2	Probiotic in water	2
G3	Probiotic in feed and water	2
NC	No probiotic	3

**After challenge**		

**Group/replicate**	**Treatment**	**Tanks**

G1_A	Probiotic in feed and challenge with *Francisella orientalis* F1	1
G1_B	Probiotic in feed and challenge with *Streptococcus agalactiae* S13	1
G2_A	Probiotic in water and challenge with *Francisella orientalis* F1	1
G2_B	Probiotic in water and challenge with *Streptococcus agalactiae* S13	1
G3_A	Probiotic in feed and water and challenge with *Francisella orientalis* F1	1
G3_B	Probiotic in feed and water and challenge with *Streptococcus agalactiae* S13	1
PC_A	No probiotic and challenge with *Francisella orientalis* F1	1
PC_B	No probiotic and challenge with *Streptococcus agalactiae* S13	1
NC	No probiotic and no challenge with bacteria	1

For the G1 group, approximately three bacterial colonies of *E. faecium* were added to 600 mL of MRS Lactobacillus Kasvibroth^®^ and incubated under agitation for 48 h at 28 °C. Thereafter, 100 mL was sprayed on 1 kg of feed (2.3 × 10^8^ CFU/g of feed), with 5 mL of universal vehicle (Vansil^®^) and dried at 28°C for 8–12 h. Fish were fed four times per day until satiety. For group G2 (aquarium volume around 27 L), 100 mL of MRS broth with cultured *E. faecium* was added to aquarium water and the water renovation was interrupted for 2 h. Thereafter, the water volume was restored. This procedure was performed every 10 days. Group G3 received both treatments.

On the day before challenge with *F. orientalis*, the water temperature was gradually decreased and maintained at 21°C (±1°C) to promote immersion infection for 3 h (7.1 × 10^5^ CFU/mL of water in the tank); this is because outbreaks of franciselose in Brazil occur in colder waters (Ortega et al., 2016). For infection with *S. agalactiae*, the water temperature was maintained at 28°C (±1°C) (Chen et al., 2012) and administered intraperitoneally at 0.1 mL/fish (8.8 × 10^5^ CFU/mL) schematic figure about isolation and treatment of fish with a probiotic bacteria. Previous data from our team showed that the immersion route is more suitable for francisellosis infection, and it is possible to establish a Lethal Dose concentration of (LD_50_). For *S. agalactiae*, only the intraperitoneally route reproduces the disease and it was possible to calculate the LD_50_ (unpublished data). In Supplementary Figure 1 represents a schematic of the isolation and administration of *E. faecium* and Supplementary Figure 2 the route of application for the challenge of pathogenic bacteria in fish.

### Growth Performance

Fish of all replicates were counted and weighed individually on the first and last day of trial. The weight gain, weight gain (%), medium final body weight, and specific growth rate (SGR) were determined (Ridha, 2006).

### Blood Sampling

Blood samples were collected at 38 days post-treatment with probiotics (16 samples per group). The fish were anesthetized with benzocaine (100 mg/L), and blood was collected by puncturing the caudal vessel in 3 mL syringes (21 G) containing 10% anticoagulant (ethylenediaminetetraacetic acid). Blood samples were used to measure hematocrit levels (Hct; %) using the microhematocrit method (Paiva et al., 2013), and red blood cells (RBCs; 10^6^/μL) were counted in a Neubauer chamber following dilution at 1:200 in Dacie solution. White blood cells (WBCs; 10^3^/μL) and total thrombocyte counts were calculated using an indirect method (Ishikawa et al., 2008). For differential counting of leukocytes, smears were stained with May–Grünwald/Giemsa/Wright stain. The hemoglobin concentration (Hgb; g/dL) was analyzed using the cyanmethemoglobin method (Collier, 1944) using commercial kits (Labtest, Lagoa Santa, MG, Brazil) to determine the hematimetric indices of mean corpuscular volume (MCV; fL) and mean corpuscular hemoglobin concentration (MCHC; g/dL).

### Innate Immune Analysis

Innate immune analysis was performed in all groups (five fish per replicate) at 38 days after treatment. Blood samples were collected without anticoagulant, allowed to coagulate, and centrifuged at 1400 × *g* for 10 min at room temperature to obtain serum, which was stored at -20°C.

Serum lysozyme activity was determined using a methodology adapted from that described by Demers and Bayne (1997). Briefly, the initial and final absorbances were measured by spectrophotometry to determine the serum lysozyme activity by the lysis of the Gram-positive bacterium, *Micrococcus lysodeikticus* (Sigma-Aldrich Chemical Co.). The reduction in absorbance of the samples was converted into an estimate of lysozyme concentration (μg mL).

Alternative complement pathway activity (ACH50) was determined using rabbit red blood cells (RaRBCs) as target cells for hemolysis, following a previously described method (Sunyer and Tort, 1995). Briefly, serially diluted sera were mixed with rabbit erythrocyte suspension and incubated at 25°C for 1 h with occasional shaking. The extent of hemolysis was estimated by measuring the optical density of the supernatant at 414 nm (OD_414_). Serum dilutions resulting in greater than 90% or less than 15% lysis were excluded from the calculation, and the serum dilution that resulted in 50% lysis of RaRBC was represented as ACH5O units/μL.

### Microbiome Analysis

After 38 days of treatment, six fish from each group were used for the bacterial microbiome analysis, and each DNA sample was isolated from the stools of two fish and pooled. The animals in each experimental group were killed with benzocaine (200 mg/L). The stool of the entire intestinal tract was removed aseptically and maintained in sterile vials with refrigeration. The samples were immediately stored in a freezer at -80°C until processing. For total DNA extraction, a commercial QIAamp DNA Stool Mini Kit (QIAGEN, Hilden, Germany) was used according to the manufacturer’s instructions. Thereafter, the V4 region of the 16S ribosomal subunit gene was amplified with primers containing overlapping regions with Illumina platform primers (Klindworth et al., 2013). After verification of the amplicon quality, the samples were sent to the Neoprospecta company for sequencing using the Illumina MiSeq platform with the 250-cycle V2 kit. The next steps were performed according to Suphoronski et al. (2019) using MOTHUR v.1.36.1 software.

### Statistical Analysis

Data were subjected to normality and homogeneity tests and subsequently to analysis of variance (ANOVA), followed by the Tukey test for comparison between arithmetic means, with a significance level of 5%. For quantitative variables that did not present a normal distribution, the non-parametric Kruskal–Wallis test was used, followed by the Dunn test with a significance level of *p* < 5%. Cumulative mortality was analyzed using the Fisher exact test with a significance level of 5% using OpenEpi v. 3.01.^1^ In metagenomics, to verify the abundance significance of taxa between groups, statistical analysis of metagenomic profiles (STAMP) was performed using parent level 1 and profile level 6 to analyze the significance between two groups using the two-sided Welch’s *t*-test (Parks et al., 2014).

## Results

### Probiotic Bacteria Selection, Genome Assembly, and Identification of Metabolic Regions

**FIGURE 1:**
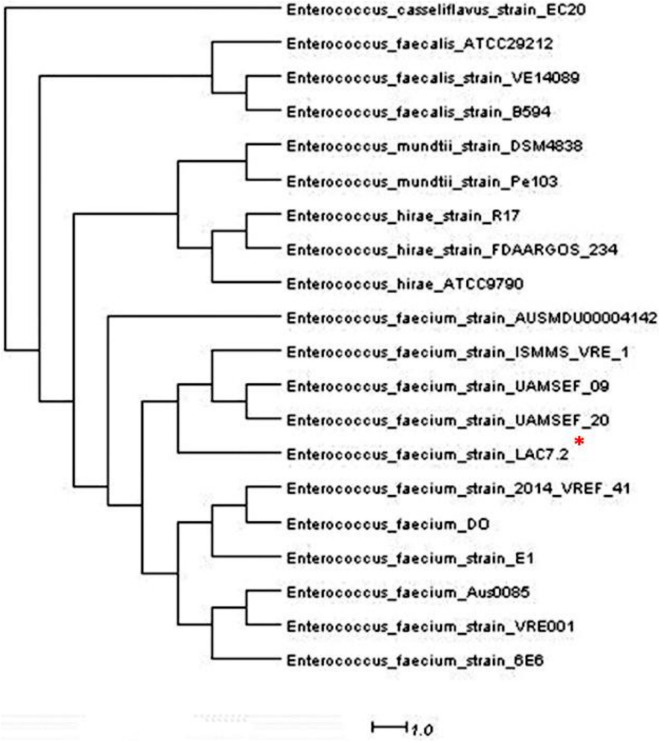
* Phylogenetic tree of *Entereococcus faeciun* strain_LAC7.2 having as predecessor the *Enterococcus casseliflavus*.

**FIGURE 2:**
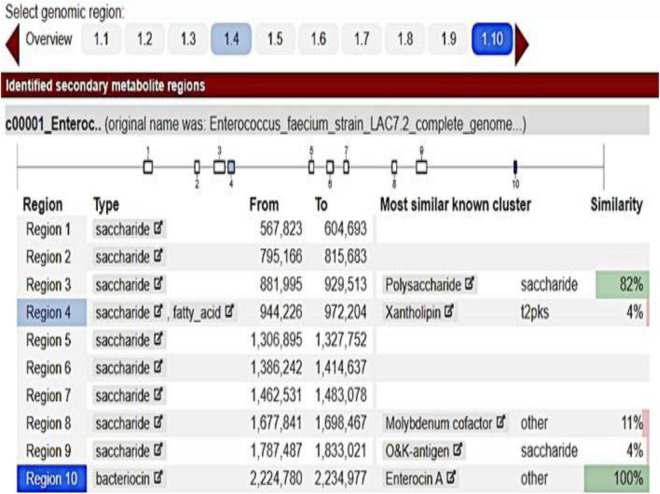
*Enterococcus faecium* anti-SMACH analysis.

The inhibition halos for the selected strains were 16 mm (*E. coli*), 15 mm (*Staphylococcus* spp.), and 10 mm (*Streptococcus* spp.) (data not shown). The inhibition zone of *F. orientalis* F1 was 18 mm. The *E. faecium* antagonism against *Staphylococcus* spp. and the inhibition of supernatant (filtered or not) of probiotic strain are demonstrated in Supplementary Figure 3. The complete genome of the *E. faecium* strain consisted of a single circular chromosome that is 2.625.745 bp in length and two plasmids of 206.375 and 80.816 bp, totaling 2.912.936 bp, 37.96% G + C content, 18 rRNA operons, 68 tRNA genes, and 131 pseudogenes (Table 1). In the phylogenetic tree, the ancestors of the bacterium were found to be *Enterococcus casseliflavus* (Figure 1). Based on the heat map, *E. faecium* was highly similar to other strains of the same species, but was not 100% similar to other strains. In the anti-SMACH analysis, the secondary metabolite, Enterocin A (Access GenBank: QIS84411.1 and locus_tag F6447_10905), was found to have 100% similarity in region 10 while polysaccharide had 82% similarity in region 3 (Figure 2). In ResFinder, two resistance genes were found in one of the plasmids: aminoglycosides and macrolides.

### Experimental Design and Basal Diet

The mean probiotic concentration was 5.53 × 10^6^ CFU/g. The final body weight and weight gain of the medium are shown in Table 3. No significant differences (*p* > 0.05) were observed in weight gain (g) (Table 3); however, the final body weight (g), SGR, and weight gain (%) were significantly higher in the G1 group than in the other groups, including the NC group.

**TABLE 3:** Growth performance of Nile tilapia due to different treatments.

**Groups**	**G1**	**G2**	**G3**	**NC**
Medium final body weight (g)	40.15 ± 1.35^a^	34.5 ± 1.03^b^	35.4 ± 0.85^b^	34.25 ± 0.90^b^
Weight gain (g)	28.64 ± 3.51	22.86 ± 0.61	22.91 ± 0.57	21.39 ± 0.9
Weight gain (%)	248.29 ± 23.3^a^	199.08 ± 3.35^b^	183.53 ± 4.23^b^	174.54 ± 0.85^b^
Specific growth rate (SGR)	3.27 ± 0.24^a^	2.88 ± 0.04^b^	2.74 ± 0.05^b^	2.65 ± 0.01^b^

*G1, probiotics in feed; G2, probiotics in water; G3, probiotics in feed and water; NC, negative control. ^a,b^Different letters indicate significant differences between the treatments (P < 0.05).*

### Blood Sampling and Innate Immune Analysis

There were no significant differences (*p* > 0.05) between the treated and control groups in the hematocrit, hemoglobin, erythrocytes, MCV, and MCHC parameters. Thrombocyte counts were significantly larger in G1 than in G2 and similar to G3. Further, there was a difference in the MCH between NC and G2. The differential leukocyte cell counts did not differ significantly between treatments for total leukocytes, neutrophils, or monocytes. No eosinophils or basophils were observed in any of the treatments tested (Table 4). The mean serum lysozyme concentrations and complement activity did not differ significantly between the treatment groups and the NC group (*p* > 0.05) (Table 4).

**TABLE 4:** Blood general parameters (mean ± SE) in the experimental groups of Nile tilapia supplemented with probiotics.

**Groups**	**G1**	**G2**	**G3**	**NC**
Hematocrit (%)	28.83 ± 0.77	21.8 ± 1.03	25.8 ± 0.89	28.6 ± 1.04
Hemoglobin (g/dL)	7.29 ± 0.41	6.49 ± 0.53	5.84 ± 0.59	5.97 ± 1.26
Erythrocytes (10^6^/μL)	1.49 ± 0.08	1.14 ± 0.04	1.18 ± 0.04	1.37 ± 0.06
Thrombocytes (10^3^/μL)	64.1 ± 3.87^a^	33.1 ± 1.33^b^	42.2 ± 3.38^a,b^	34.02 ± 3.91^b^
Leukocytes (10^3^/μL)	58.04 ± 4.61	31.88 ± 0.78	39.46 ± 2.07	50.60 ± 5.21
Lymphocytes (10^3^/μL)	25.72 ± 2.92	13.05 ± 0.52	19.48 ± 1.17	20.93 ± 1.84
Neutrophils (10^3^/μL)	28.81 ± 3.34	17.75 ± 0.71	18.45 ± 1.57	28.28 ± 3.48
Monocytes (10^3^/μL)	3.5 ± 0.47	1.07 ± 0.27	1.52 ± 0.26	1.38 ± 0.29
MCV (fL)	204.02 ± 12.37	196.32 ± 15.25	206.83 ± 12.56	216.01 ± 12.58
MCH (g/dL)	50.67 ± 2.05	56.85 ± 0.99	53.69 ± 0.82	43.71 ± 0.95
MCHC (g/dL)	27.14 ± 0.27	32.64 ± 2.66	24.25 ± 2.1	21.7 ± 1.56
Lysozyme (μg/mL)	3.33 ± 0.59	4.96 ± 1.13	3.16 ± 0.71	4.94 ± 0.80
Complement activity mean (μL for lysis of 50% of erythrocyte)	45.25 ± 2.87	52.1 ± 4.58	41.01 ± 3.09	45.63 ± 1.22

*G1, probiotics in feed; G2, probiotics in water; G3, probiotics in feed and water; NC, negative control. ^a,b^Different letters indicate significant differences between the treatments (P < 0.05).*

### Microbiome Analysis

**FIGURE 3:**
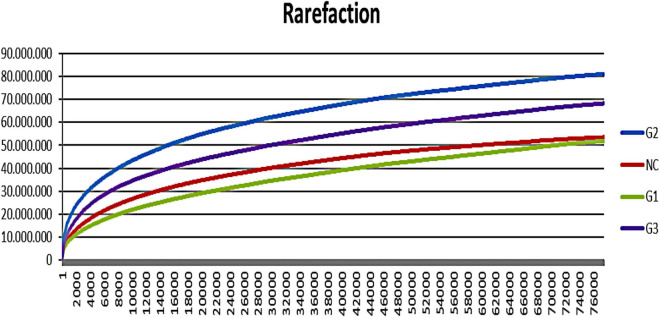
Rarefaction curve showing increasing species with the number of reads in different trial groups. G1, probiotics in feed; G2, probiotics in water; G3, probiotics in feed and water; NC, negative control.

**FIGURE 4:**
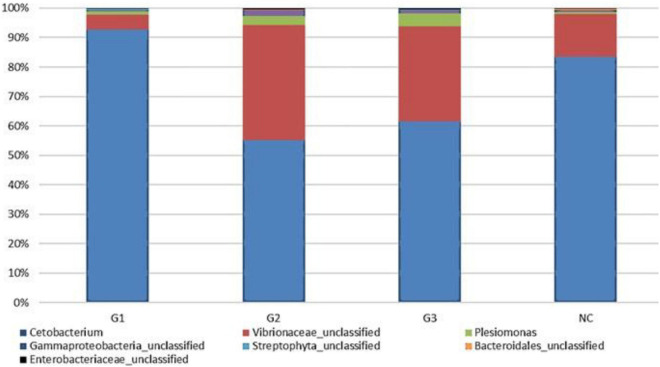
Abundance in the experimental groups and information on the percentage of sequences in each group. G1, probiotics in feed; G2, probiotics in water; G3, probiotics in feed and water; NC, negative control.

A total of 9.038.053 sequences were obtained for all groups, and 309 operational taxonomy units (OTUs) were identified. A rarefaction curve showed that sequencing was sufficient to identify most of the bacterial species present in the fish gut, suggesting that the read count of the trial was representative of the intestinal bacteriome in all groups (Figure 3). Table 5 displays the total number of sequences obtained for each group. Mothur software was used to calculate the Shannon index, which compares the diversity of species in each group. However, no significant differences were observed between groups. The G1 and NC groups presented the lowest level of species diversity, whereas the G2 and G3 groups had a higher level of diversity than the G1 and NC groups. Such finding suggests that groups receiving probiotic in water had greater diversity. The abundance of bacterial species calculated using the Mothur software is shown in Figure 4. The abundance plot displays the most abundant bacteria in each sample: *Cetobacterium*, Vibrionaceae_unclassified, *Plesiomonas*, Gammaproteobacteria_unclassified, Streptophyta_unclassified, *Bacteroidales*_unclassified, and Enterobacteriaceae_unclassified. *Cetobacterium* was most abundant in all groups; however, the G1 and NC groups had a high percentage of *Cetobacterium* compared with the G2 and G3 groups. Vibrionaceae_unclassified was more abundant in G2 and G3 than in G1 and NC.

**TABLE 5:** Number of sequences of the most abundant species in the experimental groups.

**Taxon**	**Number of sequences (%)**			
	**G1**	**G2**	**G3**	**NC**
*Cetobacterium*	91709.5 (92.7)	36749 (55.2)	54798.5 (61.7)	67008 (83.6)
Vibrionaceae_unclassified	5054.5 (5.1)	25973 (39)	28610 (32.2)	11570 (14.4)
*Plesiomonas*	1191.5 (1.2)	2020 (3)	3779 (4.3)	515 (0.6)
Gammaproteobacteria_unclassified	260.5 (0.3)	1271 (1.9)	1083 (1.2)	459 (0.6)
Streptophyta_unclassified	539 (0.5)	15.5 (0)	111.5 (0.1)	1.5 (0)
*Bacteroidales*_unclassified	36.5 (0)	188.5 (0.3)	47 (0.1)	361 (0.5)
Enterobacteriaceae_unclassified	185.5 (0.2)	303.5 (0.5)	435.5 (0.5)	227 (0.3)
Total of reads	91709.5	66520.5	88864.5	80141.5

*G1, probiotics in feed; G2, probiotics in water; G3, probiotics in feed and water; NC, negative control.*

### Mortality After Infection

**FIGURE 5:**
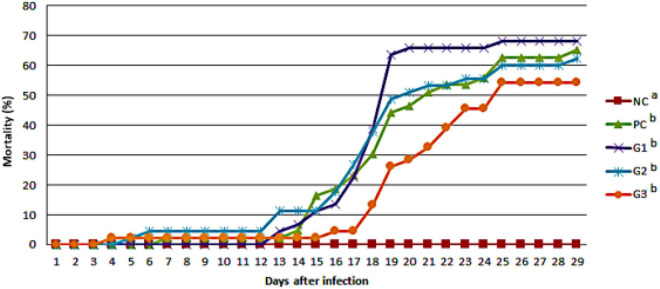
Cumulative mortality observed in the different groups after disease challenge by immersion with *Francisella orientalis*. NC, negative control (no challenge with bacteria); PC, positive control; G1, probiotics in feed; G2, probiotics in water; G3, probiotics in feed and water. ^*a,b*^Different letters indicate significant differences between treatments (*P* < 0.05).

**FIGURE 6:**
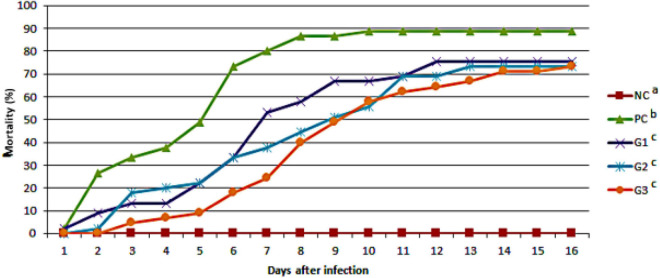
Cumulative mortality observed in the different groups after disease challenge with *Streptococcus agalactiae* administered *via* the intraperitoneal route. NC, negative control (no challenge with bacteria); PC, positive control; G1, probiotics in feed; G2, probiotics in water; G3, probiotics in feed and water. ^*a,b,c*^Different letters indicate significant differences between treatments (*P* < 0.05).

The cumulative mortality of fish infected with *F. orientalis* F1 was: PC, 65.12%; G1, 68.18%; G2, 62.15%; and G3, 54.35%. No statistical difference was found between the groups (Figure 5). However, the cumulative mortality of fish infected with *S. agalactiae* S13 showed a statistical difference between the PC and other groups that received probiotics in feed and/or water. Mortality was: PC = 88.29%; G1 = 75.56%; and G2 and G3 = 73.33% for both (Figure 6).

## Discussion

The use of probiotics in aquaculture is increasingly being considered as an eco-friendly approach to mitigate health-related problems. The disease prevention ability of probiotics is achieved through the enhancement of immunity and exclusion of pathogens (Das et al., 2013). To use a bacterium as a probiotic, tests are needed to verify its potential (Repally et al., 2018). *In vitro* tests performed on isolated Nile tilapia bacteria, *E. faecium*, revealed the potential formation of halos against pathogenic bacteria. Similar results were found by Reda et al. (2018), where antibacterial activity in Nile tilapia intestinal bacteria was observed against the pathogens *Aeromonas sobria*, *Aeromonas hydrophila*, *Pseudomonas aeruginosa*, *Pseudomonas putida*, and *Staphylococcus aureus*. Dias et al. (2019) isolated *E. faecium* from the digestive tract of juvenile neotropical ornamental cichlid fish (*Pterophyllum scalare*) and reported that we found an antagonism to *S. aureus*, *P. aeruginosa*, *E. coli*, and *A. hydrophila*, which demonstrate the probiotic potential of this bacterium in different fish species.

Of note, due to the bacterial genome used as a probiotic and with the use of bioinformatics, possible mutations, resistance genes, and antibacterial metabolites can be discovered, which can be used to define bacteria as a potential probiotic. Based on the heatmap and phylogenetic tree, strain LAC7.2 was not 100% similar to the other strains. These results could indicate the probiotic effect (i.e., better, worse, or absent). In the analysis of secondary metabolites performed *in silico*, enterocin was the main metabolite found (Figure 1); this finding aligns with that of Aymerich et al. (1996), who also observed the bactericidal power of this metabolite. Studies have shown that enterocin A has strong antimicrobial activity against *Listeria ivanovii* (Rehaiem et al., 2010) *S. aureus*, *E. coli*, and *P. aeruginosa* (Fathizadeh et al., 2020). Furthermore, this bacteriocin increases the phagocytic activity of leukocytes, beneficially influencing the animals’ immune system (Strompfová et al., 2006). This demonstrates the bactericidal capacity of this probiotic in modulating pathogenic species. Two resistance genes were found in one of the plasmids, aminoglycosides and macrolides, but whether these genes are functional remain unknown. Currently, the antimicrobials approved by Brazilian legislation for use in fish farming are florfenicol and tetracyclines [Sindicato Nacional da Indústria de Produtos para a Saúde Animal (SINDAM), 2021]; however, they do not belong to the class of antimicrobials in which the bacterium in question showed *in vitro* resistance.

Regarding fish performance, group G1 had a significant increase in final average weight and specific growth rate; however, the average weight of all groups did not differ. By evaluating juvenile rainbow trout (*Oncorhynchus mykiss*) that received different doses of *E. casseliflavus* for 8 weeks, Safari et al. (2016) found that the highest dose groups (10^8^ CFU/g of feed and 10^9^ CFU/g of feed) had significantly improved growth parameters. Such finding suggests that increasing the probiotic dose may improve performance.

In the blood analysis, thrombocyte counts were higher in the G1 group, with statistical differences found between G2 and NC. Thrombocytes are important in the organic defense mechanism, which is demonstrated by their appearance in coagulation and inflammatory processes, as well as their phagocytic activity during infection (Jatobá et al., 2011). Therefore, this result suggests that fish administered probiotics in feed have a more stimulated immune system, which is due to the presence of primary gut-associated lymphoid tissue (Panigrahi et al., 2007), than those administered probiotics in water. There was no difference in the other hematological parameters between the groups.

The proliferation of cytokines and stimulation of natural killer lymphocytes, increased production of antibodies, phagocytic rate, and lysozyme activity are responses to modulation of the immune system from probiotic supplementation (Matsuzaki and Chin, 2000). Several studies have shown that the use of probiotics in fish increases these immunological indices (Jatobá et al., 2011; Pereira et al., 2016; Ruiz et al., 2020). However, in this study, there was no difference in mean complement activity and lysozyme levels.

The microbial community of the gastrointestinal tract is known to stimulate the development of the immune system and promote competition with pathogenic microorganisms. Moreover, they are fundamental for the integrity of intestinal villi and ensure proper nutrient metabolism in fish (Hooper et al., 2012). Few studies have evaluated the intestinal community after probiotic supplementation from the metagenomic analysis. In the present study, we observed that the genus, *Cetobacterium*, varied in abundance in the G1 and NC groups, and were lower in the G2 and G3 groups than in the other groups. *Cetobacterium* is related to vitamin B12 synthesis (Tsuchiya et al., 2008) and may aid in carbohydrate degradation through symbiotic microbial activity with digestive enzymes (Pedrotti et al., 2015). In the present study, we observed that the genus *Cetobacterium* varied in abundance in groups G1 and NC, being smaller in groups G2 and G3 than in the other groups. This abundance in the G1 group is reflected in a better growth performance of this group (Table 3).

We also observed higher percentages of unclassified Vibrionaceae and *Plesiomonas* in groups G2 and G3 in the less abundant NC Plesiomonas. Such finding suggests that groups administered the probiotic only in water or water and feed had greater diversity. Standen et al. (2015) administered a commercial probiotic (AquaStar^®^), which contained various bacteria, and found different populations in the gut microbiota after 8 weeks. In NC, *Bacillus*, *Cetobacterium*, and *Mycobacterium* were the dominant genera, while *Bacillus*, *Enterococcus*, and *Pediococcus* were the largest constituents in fish fed probiotics. Previous studies have shown that fish gut communities vary within species because of factors, such as dietary input, season, developmental stage, and the surrounding habitat (Sullam et al., 2012).

Mortality caused by *S. agalactiae* S13 infection was significantly lower in all groups receiving the probiotic than in the positive control group. As observed in other studies, probiotic stimulates the immune system during infection with pathogenic bacteria (Elala and Ragaa, 2015; Safari et al., 2016). However, mortality caused by *F. orientalis* F1 did not differ between the groups. In the present study, we evaluated the administration of only one probiotic bacterium in Nile tilapia. Lee et al. (2017) carried out a study comparing the administration of different probiotics (*Bacillus subtilis* WB60 and *L. plantarum* KCTC3928) but in isolation. Few studies have verified the symbiotic effect of probiotics with other probiotics or prebiotics. Devi et al. (2019), who employed a symbiotic diet, found that immune responses in the fish were earlier than those in fish administered only probiotics or prebiotics.

Notably, the use of autoctone probiotics can benefit not only the fish itself, but also the aquatic community; this is because it is a bacterium that is already present in the environment. Probiotics isolated in other regions and countries can negatively influence the local aquatic community. The use of probiotics is directly related to unique health as with the use of fewer antimicrobials in animal production; thus, collaboration is critical to ensure there is no increase in superbugs. Therefore, further studies with autochthonous probiotic bacteria should be carried out at different concentrations and dosages to better assess their potential in fish.

The use of autochthonous probiotics can benefit the host’s microbiota as this bacterium is already present in the environment. Herein, *E. faecium* was demonstrated to be a potential probiotic for use in aquaculture as it provided a better specific growth rate and reduced the mortality of fish challenged with *S. agalactiae*.

Therefore, new studies with autochthonous probiotic bacteria should be carried out at different concentrations and dosages to better assess their potential in fish.

## Data Availability Statement

The datasets presented in this study can be found in online repositories. The names of the repository/repositories and accession number(s) can be found in the article/Supplementary Material.

## Ethics Statement

The animal study was reviewed and approved by Comissão de Ética no Uso de Animais from State University of Londrina.

## Author Contributions

SS: project administration, investigation, writing – original draft, experimental design, and data analyses. FS: investigation, visualization, and data analysis. RC: investigation, visualization, and experimental design. LM: conceptualization, data analyses, and manuscript preparation. NF: conceptualization, data analyses, manuscript preparation, and review – editing. HZ: manuscript preparation and review – editing. UP: project administration, supervision, data analysis, and manuscript preparation. All authors contributed to the article and approved the submitted version.

## Conflict of Interest

The authors declare that the research was conducted in the absence of any commercial or financial relationships that could be construed as a potential conflict of interest.

## Publisher’s Note

All claims expressed in this article are solely those of the authors and do not necessarily represent those of their affiliated organizations, or those of the publisher, the editors and the reviewers. Any product that may be evaluated in this article, or claim that may be made by its manufacturer, is not guaranteed or endorsed by the publisher.

## Footnotes

1
https://www.openepi.com/Menu/OE_Menu.htm
