# Supplementary material for: Effect of Enterococcus faecium as a Water and/or Feed Additive on the Gut Microbiota, Hematologic and Immunological Parameters, and Resistance Against Francisellosis and Streptococcosis in Nile Tilapia (Oreochromis niloticus)
Source: Front Microbiol. 2021 Oct 1;12:743957. doi: 10.3389/fmicb.2021.743957 (PMC8519173; doi:10.3389/fmicb.2021.743957)
Supplement: Supplementary file 1 [file Table_1.DOCX]

Supplementary Material

**
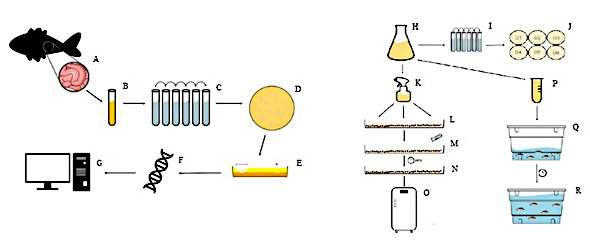
**

**Figure S.1**. **Schematic methodology for the isolation and characterization of bacterial strains with probiotic potential, and outline of the methodology used to add *Enterococcus* *faecium* LAC7.2 bacteria to food and water.** **A:** Collection from healthy tilapia intestine and feces. **B**: Collected material was seeded in MRS broth at 28ºC for 48 hours. **C:** Serial dilution of the enriched broth. **D:** Plating of dilutions in MRS agar and colony selection. **E:** Selection of colonies with better in vitro pathogen inhibition capacity (higher inhibition halo). Cross-section of spot-on-the-lawn test plate. The red bar indicates a colony that inhibited the pathogenic bacteria in the overlay. **F:** Extraction of genetic material (DNA) from the selected colony and sequencing of the complete genome. **G:** Bioinformatics analysis for prediction of genes encoding virulence factors, secondary metabolites and antimicrobial resistance. **H:** *Enterococcus* *faecium* LAC7.2 in 600mL of MRS broth at 28°C for 48 hours. **I:** Serial dilution of broth containing *Enterococcus* *faecium* LAC7.2. **J:** Seeded dilutions for counting colony-forming units/mL of broth. **K:** 100 mL of broth containing *Enterococcus* *faecium* LAC7.2 in a spray bottle **L:** 1 kg of feed distributed in a plastic container in a thin layer. Fractional spraying of 100 mL of broth containing *Enterococcus* *faecium* LAC7.2 in three steps/fractions, with homogenization between them. **M:** Addition of 5ml of universal binding vehicle (carboxymethyl cellulose) followed by homogenization. **N:** Drying of the thin layer of feed at 28ºC for 12 hours. **O:** Refrigerated storage for up to 10 days. **P:** 100 mL of broth containing *Enterococcus* *faecium* LAC7.2. **Q:** Application of the broth in the tank with 1/4 of the volume of water (25L) and homogenization by circular agitation. **R:** Restoration of water volume in the tank after 2 hours.


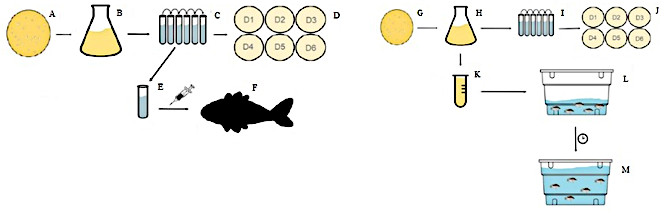


**Figure S.2. Outline of the experimental challenge with *Streptococcus agalactiae* by the intraperitoneal route, and outline of the experimental challenge with the *Francisella orientalis* by the immersion route.** **A:** Mueller Hinton agar enriched with 5% defibrinated sheep blood plate containing *Streptococcus agalactiae*. **B:** *Streptococcus agalactiae* in BHI broth at 28°C for 24 hours. **C:** Serial dilution of broth containing *Streptococcus agalactiae*. **D:** Seeded dilutions for counting colony-forming units/mL of broth. **E:** Inoculum containing 8.8x10^5 CFU/mL of *Streptococcus agalactiae*. **F:** Administration of 0.1ml/fish via the intraperitoneal route. **G:** Cystine heart agar enriched with 1% of bovine hemoglobin plate containing *Francisella orentalis*. **H:** *Francisella orientalis* in Eugon broth at 28°C for 24 hours. **I:** Serial dilution of broth containing *Francisella orientalis*. **J:** Seeded dilutions to count colony forming units/mL broth. **K:** Inoculum containing 7.1 × 10^5 CFU/ml water in the *Francisella orientalis* tank. **L:** Application of the broth in the tank with 1/4 of the volume of water (25L) and homogenization by circular agitation. **M:** Restoration of water volume in the tank after 3 hours.


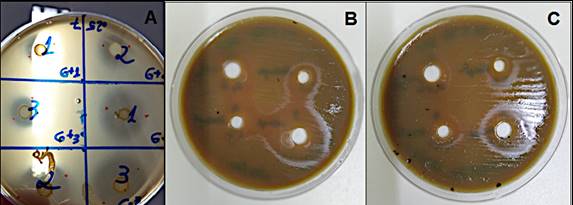


**Figure S.3. Antagonism test of *E. faecium* LAC7.2 and its supernatant against bacterial pathogens. A:** MRS agar plate containing probiotic bacteria candidate strains seeded with an overlay of Mueller-Hinton agar containing the bacterial pathogen displaying inhibition halos against *Staphylococcus* spp. The number 3 represent the 15 mm halo of *E. faecium* LAC7.2 strain against Staphylococcus spp. **B:** Inhibition halos from 15-25 mm of the probiotic bacterium supernatant in cystine heart agar enriched with 1% of bovine hemoglobin seeded with *Francisella* *orientalis.* **C:** Inhibition halos from 12-18 mm of the probiotic bacterium filtered supernatant in cystine heart agar enriched with 1% of bovine hemoglobin seeded with *Francisella orientalis.*
